# Panel of serum miRNAs as potential non-invasive biomarkers for pancreatic ductal adenocarcinoma

**DOI:** 10.1038/s41598-021-82266-5

**Published:** 2021-02-02

**Authors:** Imteyaz Ahmad Khan, Safoora Rashid, Nidhi Singh, Sumaira Rashid, Vishwajeet Singh, Deepak Gunjan, Prasenjit Das, Nihar Ranjan Dash, Ravindra Mohan Pandey, Shyam Singh Chauhan, Surabhi Gupta, Anoop Saraya

**Affiliations:** 1 Department of Gastroenterology and Human Nutrition Unit, All India Institute of Medical Sciences, Ansari Nagar, New Delhi, 110029 India; 2 Department of Biostatistics, All India Institute of Medical Sciences, New Delhi, India; 3 Department of Pathology, All India Institute of Medical Sciences, New Delhi, India; 4 Department of Gastrointestinal Surgery, All India Institute of Medical Sciences, New Delhi, India; 5 Department of Biochemistry, All India Institute of Medical Sciences, New Delhi, India; 6 Department of Reproductive Biology, All India Institute of Medical Sciences, Ansari Nagar, New Delhi, 110029 India

**Keywords:** Cancer, Molecular biology, Biomarkers, Gastroenterology

## Abstract

Early-stage diagnosis of pancreatic ductal adenocarcinoma (PDAC) is difficult due to non-specific symptoms. Circulating miRNAs in body fluids have been emerging as potential non-invasive biomarkers for diagnosis of many cancers. Thus, this study aimed to assess a panel of miRNAs for their ability to differentiate PDAC from chronic pancreatitis (CP), a benign inflammatory condition of the pancreas. Next-generation sequencing was performed to identify miRNAs present in 60 FFPE tissue samples (27 PDAC, 23 CP and 10 normal pancreatic tissues). Four up-regulated miRNAs (miR-215-5p, miR-122-5p, miR-192-5p, and miR-181a-2-3p) and four down-regulated miRNAs (miR-30b-5p, miR-216b-5p, miR-320b, and miR-214-5p) in PDAC compared to CP were selected based on next-generation sequencing results. The levels of these 8 differentially expressed miRNAs were measured by qRT-PCR in 125 serum samples (50 PDAC, 50 CP, and 25 healthy controls (HC)). The results showed significant upregulation of miR-215-5p, miR-122-5p, and miR-192-5p in PDAC serum samples. In contrast, levels of miR-30b-5p and miR-320b were significantly lower in PDAC as compared to CP and HC. ROC analysis showed that these 5 miRNAs can distinguish PDAC from both CP and HC. Hence, this panel can serve as a non-invasive biomarker for the early detection of PDAC.

## Introduction

Pancreatic ductal adenocarcinoma (PDAC) is the fourth leading cause of cancer-related deaths worldwide with a 5-year survival rate of only 9%^1^. Most of the times when the PDAC is diagnosed, it is already at an advanced stage where it cannot be resected. Hence, there is an urgent need to find biomarkers for the early detection of PDAC.

MicroRNAs (miRNAs) are a subset of small non-coding RNAs that regulate gene expression at the post-transcriptional level by binding to the 3ʹ untranslated region (UTR) of target mRNAs^2^. Altered expression of miRNAs has been shown to be involved in the regulation of crucial pathological processes in tumorigenesis, progression and metastasis^3–5^. Aberrant expression of miRNAs has been detected in a wide variety of cancers including breast, lung, colorectal, hepatocellular and even pancreatic cancer^6–10^. Several groups have shown that expression of miR-21, miR-155, miR-196a and miR-222 increases in pancreatic tumour tissue ^11–14^. Recent studies have looked at the possibility of using these miRNAs as possible diagnostic and prognostic markers^15,16^. Several reports indicate that miRNA expression profiles could be useful in the diagnosis of specific cancer types^17,18^.

It has been well established that serum contains a large number of stable miRNAs derived from various tissues/organs. Highly expressed miRNAs in specific tissues may leak into circulation as biomarkers for tissue injuries, and recent studies have shown the utility of miRNAs as biomarkers for cancers like pancreatic, colorectal, lung, prostate cancer etc^19–23^. A few studies have profiled the plasma miRNAs in PDAC and even shown their clinical significance ^19,24^.

A large, retrospective cohort study in which patients had chronic pancreatitis (CP) of at least five years duration before being diagnosed with PDAC found a 14-fold increased risk in these patients^25^. Since CP is a known risk factor for the development of PDAC; it seems a promising idea to screen the CP patients for the malignant transformation associated phenotypic changes. However, till date, no blood-based marker has been shown to be useful in the early detection of PDAC in this high-risk, asymptomatic population. Early diagnosis for PDAC requires markers with high sensitivity and specificity. The standard serum marker, sialylated Lewis blood group antigen CA19-9, is widely used, but its use is limited to monitoring responses to therapy and not as a diagnostic marker^26^.

To the best of our knowledge, no previous study has used Next Generation Sequencing (NGS) to identify miRNAs expressed in CP. Hence, in the present study, both high-throughput NGS and quantitative reverse transcription PCR (qRT-PCR) assays were used to characterize the miRNA expression profile in FFPE tissue and serum from PDAC and CP patients. The goal was to identify a panel of serum miRNAs that could serve as a diagnostic biomarker for the early detection of PDAC in a high-risk group like CP patients.

## Results

### Selection of candidate miRNAs from the NGS data

NGS analysis was performed to identify the differentially expressed miRNAs in 27 PDAC, 23 CP, and 10 normal pancreatic tissue specimens from autopsy cases. Results from PDAC and CP groups were compared with the normal pancreas. A miRNA was considered differentially expressed when it had both fold change ≥ 2.0 or ≤ 0.5 and a p-value < 0.05. Based on these criteria, 219 miRNAs were identified to be differentially expressed between patients with PDAC and CP, including 120 up-regulated miRNAs and 99 down-regulated miRNAs. From the list of differentially expressed miRNAs, 8 miRNAs were selected for further validation with qRT-PCR—four up-regulated miRNAs (miR-215-5p, miR-122-5p, miR-192-5p and miR-181a-2-3p) with the highest fold change between PDAC and CP, and the four most down-regulated miRNAs (miR-30b-5p, miR-216b-5p, miR-320b, and miR-214-5p) in PDAC versus CP (Table 1).

**Table 1:** List of 8 most differentially expressed miRNAs between PDAC and CP selected from NGS data.

S. No	miRNA	Average NGS read counts (reads per million)		Fold change	Regulation
		PDAC expression	CP expression		
1	miR-122-5p	29.184	0.617	47.256	Up
2	miR-181a-2-3p	79.163	3.9201	20.1903	Up
3	miR-215-5p	1051.868	62.665	16.785	Up
4	miR-192-5p	36,052.908	4321.683	8.342	Up
5	miR-320b	1.312	8.264	0.257	Down
6	miR-216b-5p	6.331	27.819	0.227	Down
7	miR-214-5p	2.507	11.281	0.222	Down
8	miR-30b-5p	2.787	37.094	0.075	Down

### Validation of tissue miRNA expression by qRT-PCR

**Figure 1:**
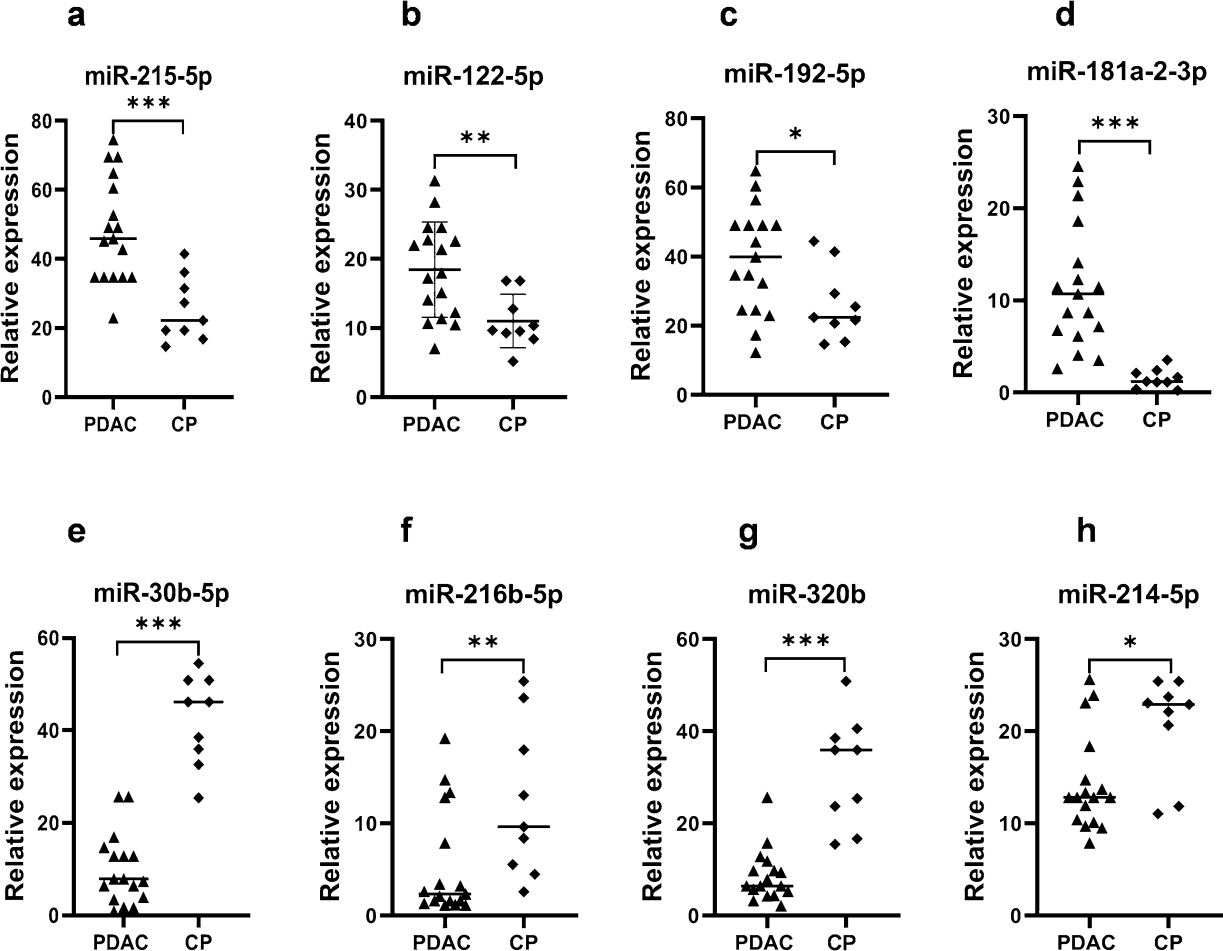
Relative expression of miRNAs in PDAC FFPE tissue samples compared with that in CP FFPE tissues. (**a**–**d**) Up-regulated miRNAs. (**e**–**h**) Down-regulated miRNAs. *p < 0.05, **p < 0.001, ***p < 0.0001.

To validate the NGS results, the expression levels of the eight selected miRNAs were measured by qRT-PCR in 26 FFPE tissue samples, which included 17 PDAC cases and 9 CP samples (the same samples used in the NGS). As shown in Fig. 1a–d, four miRNAs including miR-215-5p (p < 0.0001), miR-122-5p (p < 0.001), miR-192-5p (p < 0.05) and miR-181a-2-3p (p < 0.0001) were significantly up-regulated in PDAC tissue compared to the CP, consistent with the results of the NGS. The other four selected miRNAs, namely, miR-30b-5p (p < 0.0001), miR-216b-5p (p < 0.001), miR320b (p < 0.0001) and miR-214-5p (p < 0.05), were significantly down-regulated in PDAC compared to CP (Fig. 1e–h).

### Levels of miRNAs in serum samples by qRT-PCR

**Figure 2:**
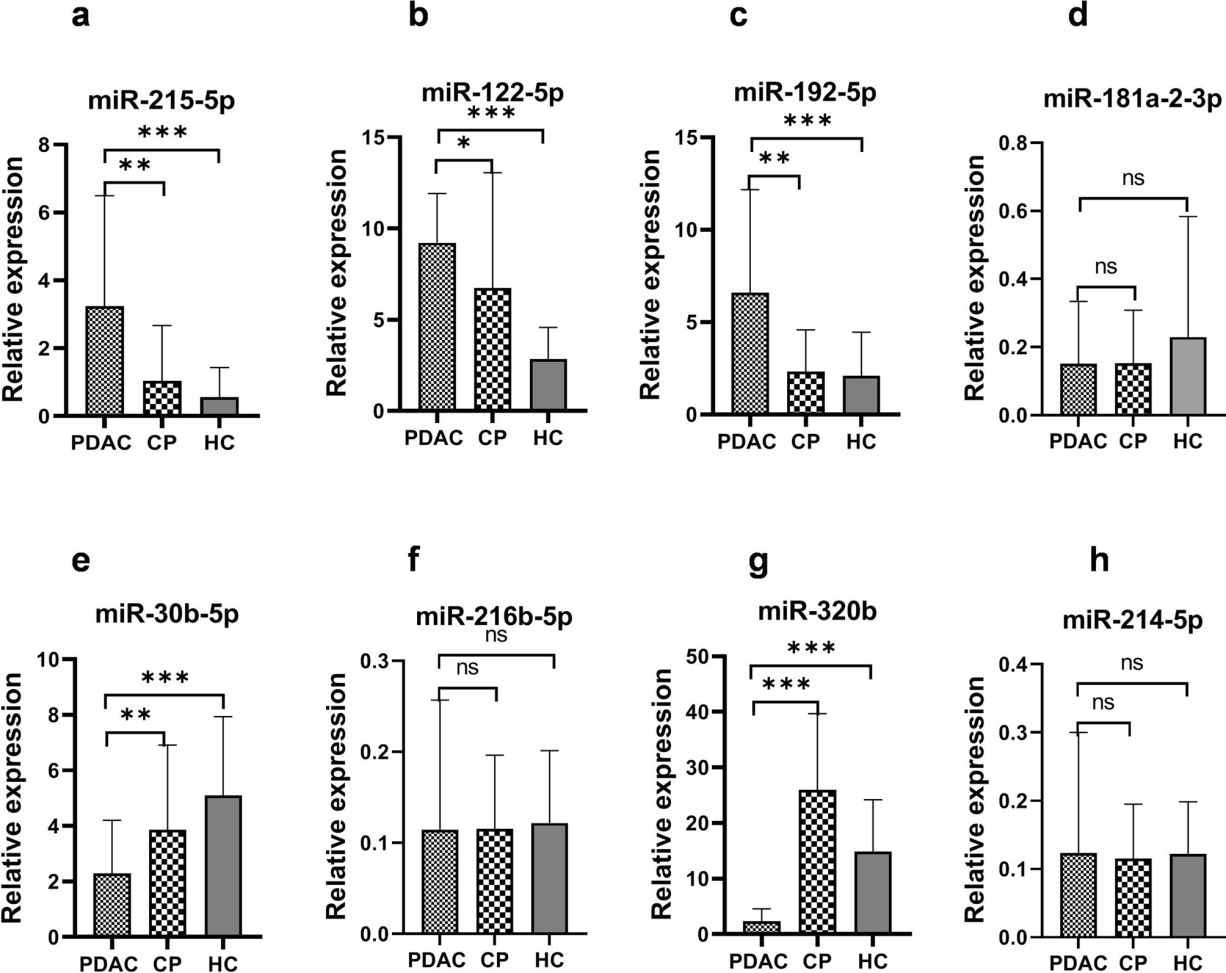
Relative levels of up-regulated miRNAs in serum of PDAC compared with that in CP and HC (**a**–**d**) and down-regulated miRNAs in serum of PDAC compared with that in CP and HC (**e**–**h**). *p < 0.01, **p < 0.001, ***p < 0.0001.

The levels of the eight selected miRNAs were measured in 125 serum samples (50 PDAC, 50 CP, and 25 HC) using qRT-PCR. The levels of these miRNAs were then compared among the three groups, including the comparisons between PDAC and CP, and between PDAC and HC. As shown in Fig. 2a–c, three miRNAs including miR-215-5p (*P* < 0.001), miR-122-5p (*P* < 0.01), and miR-192-5p (*P* < 0.001) were significantly up-regulated in the serum of PDAC patients compared to that of CP patients. Similarly in comparing the PDAC and HC samples, the same three candidate miRNAs (miR-215-5p, miR-122-5p and miR-192-5p) had significantly up-regulated levels (p < 0.0001 for each) in the serum of PDAC patients than in the HC (Fig. 2a–c). However, the serum levels of miR-181a-2-3p, exhibited no significant difference among the three studied groups (p = 0.45, 0.38 and 0.56; Fig. 2d) even though its tissue expression was much higher in PDAC (Fig. 1d).

Regarding the miRNAs that are down-regulated in PDAC, serum from patients with PDAC showed significantly decreased levels of miR-30b-5p (p < 0.001) and miR-320b (p < 0.0001) (Fig. 2e,g), whereas no significant difference was observed in miR-216b-5p (p = 0.766) and miR-214-5p (p = 0.823) levels between the PDAC and CP (Fig. 2f and h).

As shown in Fig. 2a–c, the circulating levels of miR-215-5p, miR-122-5p and miR-192-5p in serum samples gradually increased from HC to CP to PDAC, suggesting their role in tumor progression. It is noteworthy that the difference in levels from HC to CP was not significant; however, between CP and PDAC, the enhancement in their levels became highly significant (Fig. 2a–c). Furthermore, the levels of miR-30b-5p in serum gradually decreased from HC to CP to PDAC, suggesting its role as a tumor suppressor (Fig. 2e).

Further assessment of the expression levels of miR-215-5p, miR-122-5p and miR-192-5p from FFPE tissue samples (Fig. 1a–c) showed an overall upregulation in PDAC tissues which correlated well with the higher levels in PDAC serum samples (Fig. 2a–c). Similarly, the expression levels of miR-30b-5p and miR-320b from FFPE tissue samples (Fig. 1e and g) showed an overall downregulation in FFPE PDAC tissue samples and also in PDAC serum samples (Fig. 2e and g). The serum levels of these miRNAs replicated the expression level patterns seen in FFPE tissues suggesting that these miRNAs could serve as a non-invasive biomarker for the early detection of PDAC.

The tissue and serum levels of the miRNAs were also analyzed according to the stage of PDAC (Tables 2 and 3). The results showed that six of the miRNAs were significantly different between FFPE tissues of CP and early stages of PDAC (Table 2). However, miRNA levels were not different when comparing early stages of PDAC with advanced stages. Similarly, in serum samples of CP and early stages of PDAC, five of the miRNAs showed significantly different levels (Table 3). This suggests that these miRNAs may be relevant for early detection of PDAC.

**Table 2:** Comparison of relative expression of miRNAs in FFPE tissue samples of CP and different stages of PDAC.

miRNA	Relative expression in FFPE tissue mean +/− SD			p-value comparing	
	CP (n = 9)	PDAC Stage I + II (n = 4)	PDAC Stage III + IV (n = 13)	CP with PDAC stage I + II	PDAC stage I + II with PDAC stage III + IV
miR-215-5p	25.451 ± 9.264	49.909 ± 17.859	47.787 ± 14.915	0.033	0.671
miR-122-5p	11.00 ± 3.863	22.406 ± 7.912	17.262 ± 6.445	0.012	0.231
miR-192-5p	26.206 ± 10.573	41.552 ± 19.658	38.497 ± 14.796	0.029	0.764
miR-181a-2-3p	1.532 ± 1.040	14.136 ± 9.539	15.672 ± 8.281	0.044	0.986
miR-30b-5p	42.349 ± 9.731	15.187 ± 9.143	14.332 ± 9.940	0.032	0.886
miR-216b-5p	4.308 ± 8.348	4.537 ± 5.531	5.630 ± 6.183	0.653	0.822
miR-320b	31.473 ± 11.837	7.697 ± 2.565	8.635 ± 6.430	0.001	0.991
miR-214-5p	20.702 ± 5.447	17.028 ± 4.781	16.254 ± 7.158	0.059	0.729

**Table 3:** Comparison of relative expression of miRNAs in serum samples of CP and different stages of PDAC.

iRNA	Relative expression in Serum mean +/− SD			p-value comparing	
	CP (n = 50)	PDAC Stage I + II (n = 14)	PDAC Stage III + IV (n = 36)	CP with PDAC stage I + II	PDAC stage I + II with PDAC stage III + IV
miR-215-5p	1.033 ± 1.638	5.365 ± 5.537	6.196 ± 6.189	0.047	0.651
miR-122-5p	6.732 ± 6.318	11.868 ± 10.561	12.959 ± 13.742	0.050	0.771
miR-192-5p	2.320 ± 2.258	7.914 ± 6.788	6.843 ± 6.570	0.044	0.868
miR-181a-2-3p	0.173 ± 0.264	0.153 ± 0.210	0.165 ± 2.281	0.986	0.895
miR-30b-5p	3.998 ± 3.048	0.211 ± 2.933	0.884 ± 3.940	0.036	0.587
miR-216b-5p	0.115 ± 0.180	0.114 ± 2.297	0.121 ± 1.659	0.561	0.771
miR-320b	25.976 ± 13.702	5.251 ± 5.252	4.347 ± 4.165	0.050	0.499
miR-214-5p	0.113 ± 0.197	0.152 ± 0.220	0.112 ± 0.158	0.446	0.138

### Diagnostic value of the differentially expressed miRNAs for PDAC

**Figure 3:**
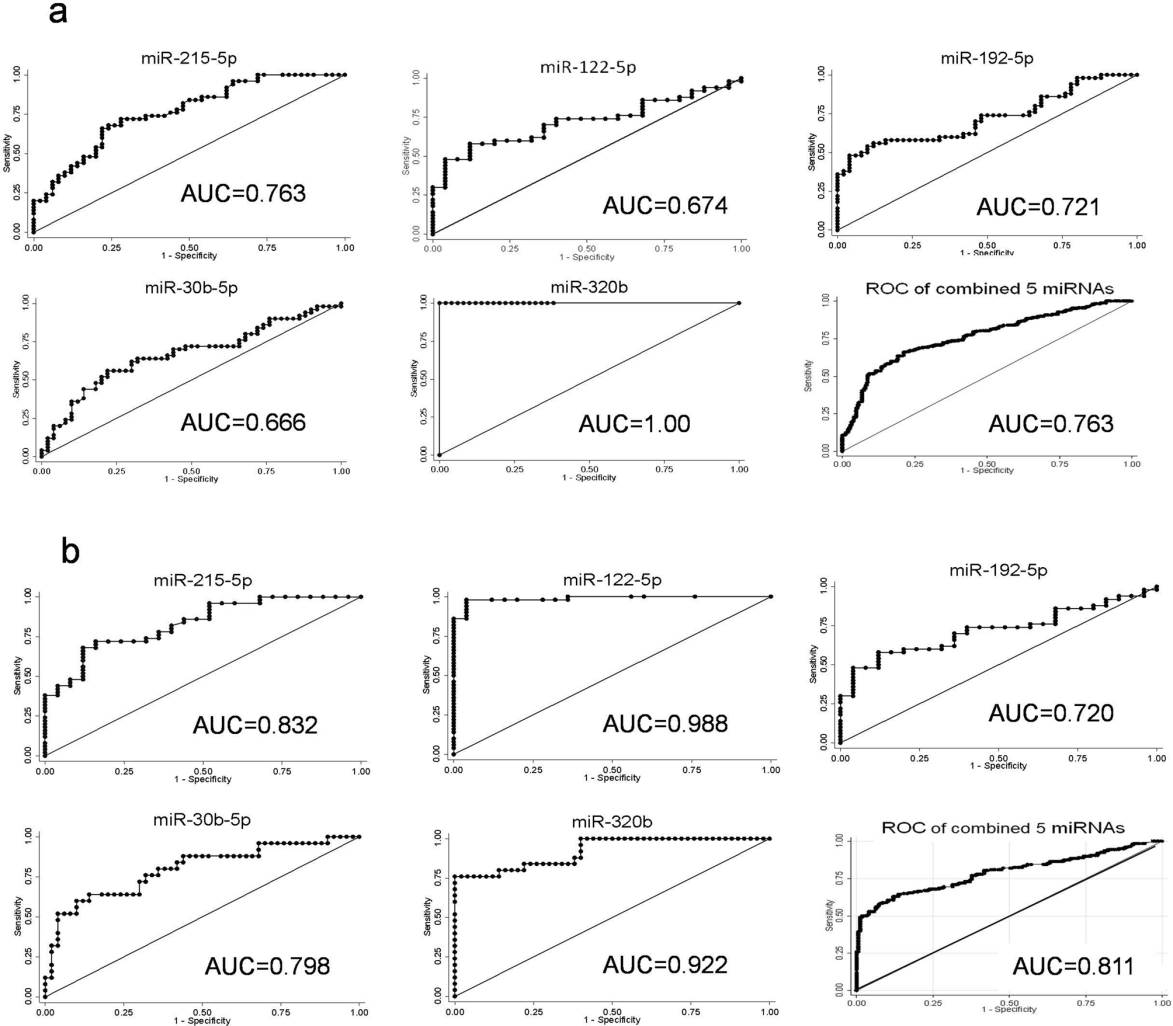
Receiver operating characteristic (ROC) curves of serum miRNAs based on results obtained by qRT-PCR (**a**) patients with PDAC versus CP, (**b**) patients with PDAC versus HC.

It was observed that the relative levels of five miRNAs in the serum of the PDAC patients were significantly different from those of the CP patients and HC (Fig. 2), which was similar to the NGS results. Their diagnostic capability to serve as biomarkers for early detection of PDAC was checked by making receiver operating characteristic (ROC) curves and calculating the area under curve (AUC), sensitivity and specificity for each miRNA. The results demonstrated that all the five significant miRNAs (miR-215-5p, miR-122-5p, miR-192-5p, miR-30b-5p, and miR-320b) could potentially discriminate patients with PDAC from CP and HC (Fig. 3a,b). Among these miRNAs, miR-320b showed the highest diagnostic accuracy for distinguishing PDAC patients from CP with an AUC of 1.00; 95% CI: 0.002–0.054 (Fig. 3a and Table 4). MiR-215-5p (95% CI: 0.664–0.839), miR-192-5p (95% CI: 0.621–0.805), miR-122-5p (95% CI: 0.568–0.760) and miR-30b-5p (95% CI: 0.568–0.760) showed a moderate discrimination with AUC value less than 0.8 (Fig. 3a and Table 4). In addition, serum levels of miR-122-5p, miR-320b and miR-215-5p showed a high discrimination for PDAC from HC with AUC value of 0.988 (95% CI: 0.927–0.999), 0.922 (95% CI: 0.833–0.970) and 0.832 (95% CI: 0.721–0.904) respectively (Fig. 3b and Table 5).

**Table 4:** Diagnostic performance of the five selected serum miRNAs in discriminating PDAC patients from chronic pancreatitis. *AUC, area under the curve; CI, confidence interval.

miRNA	AUC	95% CI	Cut-off point	Sensitivity %	Specificity %
miR-215-5p	0.763	0.664–0.839	0.744	72	72
miR-122-5p	0.674	0.568–0.760	5.834	98	56
miR-192-5p	0.721	0.621–0.805	2.055	70	54
miR-30b-5p	0.666	0.568–0.760	1.924	70	56
miR-320b	1.00	0.002–0.054	8.631	100	100

**Table 5:** Diagnostic performance of the five selected serum miRNAs in discriminating PDAC patients from healthy controls. *AUC, area under the curve; CI, confidence interval.

miRNA	AUC	95% CI	Cut-off point	Sensitivity %	Specificity %
miR-215-5p	0.832	0.721–0.904	0.611	72	72
miR-122-5p	0.988	0.927–0.999	5.834	98	96
miR-192-5p	0.720	0.604–0.817	1.669	74	60
miR-30b-5p	0.798	0.691–0.883	2.741	76	68
miR-320b	0.922	0.833–0.970	4.168	84	78

Further ROC curve analyses were performed for combinations of miR-215-5p, miR-122-5p, miR-192-5p, miR-30b-5p, and miR-320b. The AUC of the five-miRNA panel was 0.763 (95% CI, 0.724–0.800; Fig. 3a) for distinguishing PDAC from CP and 0.811 (95% CI, 0.744–0.829; Fig. 3b) for distinguishing PDAC from HC.

## Discussion

Early diagnosis of PDAC presents a challenge due to the lack of sensitive and specific biomarkers; hence, there is a need for new early diagnostic tools. Recent studies by Jin et al.^27^ and others^19,26,28,29^ have demonstrated that miRNAs are stable in serum/plasma and hence, can be readily detected by various assays such as NGS technology, miRNA microarray, and qRT-PCR. More importantly, the unique serum/plasma miRNA expression profiles for pancreatic cancer may serve as fingerprints for their detection^19,26,28,29^. A serum miRNA–based biomarker associated with pancreatic cancer could possibly allow detection of the tumors without invasive procedures such as biopsy and surgery. In this study, the expression levels of tissue miRNAs in PDAC, CP and normal pancreas were determined by NGS. Eight differentially expressed miRNAs were selected and their levels were quantified in serum of PDAC, CP patients and healthy controls by qRT-PCR. The results showed that 5 serum miRNAs; three up-regulated (mir-215-5p, mir-122-5p, and mir-192-5p) and two down-regulated (mir-30b-5p and mir-320b) have strong potential as a biomarker for the early detection of PDAC.

In the current study, a significant increase was observed in the expression of miR-215-5p, miR-122-5p and miR-192-5p in PDAC tissues compared to both CP tissue and normal pancreatic tissue using two different techniques (NGS and qRT-PCR). This suggests their involvement in tumour progression; however, there are conflicting reports in the literature about the role of these miRNAs in various cancers. The expression of miR-215-5p was reported to be up-regulated in gastric cancer and gliomas^30,31^; with higher miR-215-5p expression associated with higher-grade of glioma^32^. But reports in other cancer tissues suggest that it serves as a tumor suppressor^33^. Similarly, a lot of studies have looked at the expression of miR-122-5p in cancer tissues with most of them showing a tumor suppressor role for miR-122-5p. Most studies in pancreatic cancer and hepatocellular carcinoma show that miR-122-5p is underexpressed in tumor tissue^15,24,34–37^. However, studies in renal cell carcinoma and colorectal cancer show overexpression of this miRNA in the tumor tissues^38,39^. Likewise, controversial results have been reported about the alteration of miR-192-5p in PDAC tissue specimens. While Flammang et al.^40^ and Botla et al.^41^ reported lower expression of miR-192-5p in PDAC tissue; others have reported increased expression in pancreatic cancer tissue^24,42,43^. In fact, ectopic expression of miR-192-5p associates with enhanced cell proliferation and migration, reduced apoptosis and promotes cell cycle progression in pancreatic cancer^43^.

Interestingly, studies measuring the circulatory levels of all these three miRNAs show higher levels in cancer patients compared to controls, which is in concordance with data in the present study. At the blood level, it has been shown that serum miR-215-5p has a relatively high value as a promising biomarker in diagnosing hepatocellular carcinoma ^44^ and osteosarcoma ^45^. In keeping with our findings, different studies identified the up-regulation of miR-122-5p in plasma or whole blood of patients with PDAC^24,46,47^. Mazza et al. found that though plasma miR-122-5p levels had low specificity as a diagnostic marker with no significant difference between levels in CP and PDAC patients but it could serve as an independent negative prognostic factor as it significantly associated with tumor stage and metastasis^47^. Studies in other cancer types like hepatocellular carcinoma and colorectal cancer also show increased serum levels of miR-122-5p^38,48^. Levels of miR-192-5p have also been reported to be higher not only in serum or plasma of pancreatic cancer patients but also in the serum of esophageal squamous cell carcinoma (ESCC) patients ^24,42,49^.

In contrast to the conflicting literature regarding these upregulated microRNAs, the available reports about the role of miR-30b-5p and miR-320b in cancer are more consistent. The lower expression of both these microRNAs in the present study indicated that they act as tumor suppressors in PDAC. Indeed, miR-30b-5p is found to be down-regulated in numerous human cancers, including PDAC^50^. Qin et al. analyzed the expression of miR-30b-5p in 90 patients of HCC and found that expression of miR-30b-5p was down-regulated in HCC tissue, thus inferring that miR-30b-5p acts as a tumor suppressor^51^. Moreover, another study showed that the expression level of miR-30b-5p in 32 ESCC tissues was significantly lower than that in adjacent normal tissues^52^. Liu et al. also found that miR-30b-5p functions as a tumor suppressor in renal cell carcinoma by targeting cell proliferation, metastasis and epithelial-to-mesenchymal transition^53^. As a member of the miR320 cluster, miRNA-320b has also been identified to play a suppressive role in various tumors, including colorectal cancer^54^, glioma^55^ and nasopharyngeal carcinoma^56^. Tadano et al. found that miR-320 family, which inhibits cell proliferation, is frequently down-regulated in colorectal adenoma and submucosal invasive carcinoma tissues^57^. A recent study showed that transfection of pancreatic cancer cell lines with miR-320b mimics decreased their proliferation and invasion ability indicating that miR-320b has a tumor-suppressive role^58^.

Since, the present study revealed that the expression pattern of miR-215-5p, mir-122-5p and miR-192-5p is gradually increasing from HC to CP to PDAC (Fig. 2) while the levels of miR-30b-5p in serum gradually decreased from HC to CP to PDAC, hence, monitoring their serum levels at the stage of CP could be an effective marker for the early detection of PDAC.

Another interesting observation from this study is that pattern of serum miRNA levels does not necessarily correspond to their expression pattern in the tissue. In the present context, expression of miR-181a-2-3p, miR-216b-5p and miR-214-5p were significantly different in PDAC tissue compared to CP tissue by both NGS and qRT-PCR but there was no significant difference in their serum levels between the two groups of patients. This observation is not unique to this study as many other reports show little or no concordance between tissue and circulatory levels of the same microRNA^24,40^. This indicates that due care should be given to determine the miRNA levels in both tissue and serum as miRNAs showing concordance would serve as better biomarkers.

## Conclusion

In conclusion, this study identified 5 serum miRNAs (miR-215-5p, miR-122-5p, miR-192-5p, miR-30b-5p and miR-320b) that could differentiate PDAC cases from CP and healthy controls with a high degree of accuracy. Thus, these identified miRNAs could potentially function as early non-invasive diagnostic biomarkers of PDAC. Further studies are needed to validate these findings with larger sample size. Future investigations could also explore the molecular mechanism employed by these miRNAs in the progression of pancreatic ductal adenocarcinoma.

## Materials and methods

### Ethics statement

This study was approved by the Institutional Ethics Committee, All India Institute of Medical Sciences, New Delhi, India. Written informed consent was obtained from participants for the use of their tissues and blood samples in this study. All methods were performed in accordance with the relevant guidelines and regulations.

### FFPE tissue and serum sample collection

Formalin-fixed paraffin-embedded (FFPE) tissue blocks from each group: 27 PDAC patients; 23 CP patients who underwent surgery for pain relief; and normal pancreatic tissue specimens from 10 autopsy cases were included for comparison purposes and used for miRNA profiling by NGS. The serum samples from 50 PDAC patients; 50 CP patients and 25 healthy controls (HC) were processed and stored in -80 °C until further experiments. PDAC was diagnosed on the basis of radiological or histopathological examinations. CP was diagnosed on the basis of clinical diagnostic criteria and radiological examinations. The demographic and clinical variables of the PDAC and CP patients are shown in Tables 6 and 7, respectively. Samples were collected from the Department of Gastroenterology and Department of Pathology, All India Institute of Medical Sciences, New Delhi, India.

**Table 6:** Clinical characteristics of the PDAC patients (n = 50).

Characteristics	PDAC (%)
Age (Mean ± SD) years	55.65 ± 11.67
**Sex**	
Male	35(70%)
Female	15(30%)
Abdominal pain	43 (86%)
Jaundice	26 (52%)
Anorexia	40 (80%)
Weight Loss	39(78%)
Diabetes Mellitus	13 (26%)
Locally advanced disease	18 (36%)
Lymph node involvement	27 (54%)
Vascular encasement	29 (58%)
Metastasis	17 (34%)

**Table 7:** Clinical characteristics of the CP patients (n = 50).

Characteristics	CP (%)
Age (Mean ± SD) Years	34.02 ± 10.35
**Sex**	
Male	37 (74%)
Female	13 (26%)
Disease Duration (Mean ± SD) Months	55.96 ± 25.41
Follow-Up Duration (Mean ± SD) Months	37.04 ± 31.37

### RNA isolation from FFPE tissue blocks and serum samples

One FFPE block was selected from each patient for miRNA analysis. From each of these blocks, three 10-µm sections were cut and taken on fresh slides; the tumour area was marked and micro-dissected after deparaffinization. The tissue from 3 slides (of the same block) was scraped into a 2 ml tube and mixed with the lysis buffer provided in the miRCURY RNA Isolation Kit (Tissue) from Exiqon (Exiqon, USA). The rest of the protocol was according to the instructions provided in the kit. The integrity of extracted RNA samples was analyzed using Agilent Bioanalyzer 2100 (Agilent Technologies, CA, USA).

Total RNA was extracted and purified from 500 μl of serum using the miRCURY RNA isolation kit – Biofluids (Exiqon, Denmark) following the manufacturer’s instructions. Finally, the RNA was eluted in 50 μl volume.

### Library construction and next-generation sequencing

Small RNA libraries were constructed using the Illumina Sample Preparation Kit, following the TruSeq Small RNA Sample Preparation Guide (Illumina, CA, USA)^59^. Approximately 500 ng of total RNA was used. The 3′ adaptors were ligated to the specific 3′OH group of miRNAs followed by 5′ adaptor ligation. The ligated products were reverse transcribed (Superscript III, Invitrogen, Whitefield, Bangalore) and amplified by PCR (17 cycles). The amplified libraries were size selected in the range of 140 bp–160 bp followed by overnight gel elution and salt precipitation^60^. Finally, the sequencing library was quantified by qRT-PCR using the Kapa Library Quantification Kit (Kapa Biosystems, Wilmington, MA, USA)^61^. The qPCR quantified library was subjected to sequencing on an Illumina sequencer (NextSeq500, Illumina) for 75 bp single-end chemistry.

### Bioinformatics analysis of miRNA sequencing data

The adapter sequences and low-quality sequences were removed prior to data analysis^62^. Sequences >  = 16 bp and <  = 35 bp length were considered for further analysis. All the sequences were aligned to the Homo sapiens genome using bowtie-1.1.1. Aligned reads were extracted and checked for ncRNA (rRNA,tRNA,snRNA and snoRNA) contamination. The unaligned reads to ncRNAs were clustered based on the 100% coverage and similarity to generate the read count. Clustered reads were used for Known miRNA prediction.

Further, homology search of these miRNAs was done against all matured Homo sapiens miRNA sequences retrieved from miRbase-21 database using NCBI-blast-2.2.30 + . Known miRNAs and the read usage statistics reports were generated. DGE (Differential gene expression) analysis was carried out using DESeq tool. A miRNA was considered differentially expressed when the miRNA had both a fold change of ≥ 2 or ≤ 0.5 and a P-value < 0.05. Only differential miRNAs with high abundance reads were selected for further investigation.

### Validation of miRNA expression by real-time PCR

The expression levels of the eight candidate miRNAs (miR-215-5p, miR-122-5p, miR-192-5p, miR-181a-2-3p, miR-30b-5p, miR-216b-5p, miR-320b, and miR-214-5p) selected from the NGS data were measured by qRT-PCR. 6 μl of total RNA was converted to cDNA in a 20 μl reaction mixture using the miRCURY LNA Universal cDNA Synthesis Kit (Exiqon, Denmark) following the manufacturer’s instructions. Quantification by real-time PCR was performed on Stratagene Mx3005P thermal cycler (Agilent Technologies, USA), using 4 μl of 1:40 diluted cDNA per tube along with the Exilent SYBR miRNA assay kits (Exiqon, Denmark), and LNA based specific primer assays (Qiagen, Germany). MxPro qRT-PCR software was used for the generation of the data. The miRNA expression was assayed in triplicate. Normalization of the miRNA expression levels was done using U6 as a reference control for tissue miRNAs and UniSp6 as an exogenous control for serum miRNAs. Results were analyzed by checking the relative expression of miRNAs by the 2^−ΔCT^ method.

### Statistical analysis

Statistical analysis was performed using STATA 14.0 statistical software (Texas, USA) and graphs were generated using GraphPad Prism 8.0. One-way ANOVA test was used to evaluate the differential expression of serum miRNA levels between PDAC, CP patients and healthy individuals. The significance of tissue miRNA expression levels was determined by Mann–Whitney test. The area under the ROC curves (AUC) generated by STATA 14.0 was used to evaluate the performance of the selected miRNAs in discriminating the PDAC from CP and HC. Sensitivity and specificity were calculated at an optimal cutoff point to determine the accuracy of differentially expressed miRNAs. A p-value < 0.05 was considered to be statistically significant.
